# Long-term outcomes following first short-term clinically important deterioration in COPD

**DOI:** 10.1186/s12931-018-0928-3

**Published:** 2018-11-20

**Authors:** Ian P. Naya, Lee Tombs, Hana Muellerova, Christopher Compton, Paul W. Jones

**Affiliations:** 1 Respiratory Medicine, GSK, Brentford, Middlesex, UK; 2 Precise Approach Ltd, Contingent worker on assignment at GSK, Uxbridge, Middlesex UK

**Keywords:** Clinically important deterioration, Composite measures, COPD, Mortality

## Abstract

**Background:**

Chronic obstructive pulmonary disease (COPD) is characterized by varying trajectories of decline. Information regarding the prognostic value of preventing short-term clinically important deterioration (CID) in lung function, health status, or first moderate/severe exacerbation as a composite endpoint of worsening is needed. We evaluated post hoc the link between early CID and long-term adverse outcomes.

**Methods:**

CID was defined as ≥100 mL decrease in forced expiratory volume in 1 s (FEV_1_), ≥4-unit increase in St George’s Respiratory Questionnaire (SGRQ) score from baseline, and/or a moderate/severe exacerbation during enrollment in two 3-year studies. Presence of CID was assessed at 6 months for the principal analysis (TORCH) and 12 months for the confirmatory analysis (ECLIPSE). Association between presence (+) or absence (-) of CID and long-term deterioration in FEV_1_, SGRQ, future risk of exacerbations, and all-cause mortality was assessed.

**Results:**

In total, 2870 (54%; TORCH) and 1442 (73%; ECLIPSE) patients were CID+. At 36 months, in TORCH, CID+ patients (vs CID-) had sustained clinically significant worsening of FEV_1_ (- 117 mL; 95% confidence interval [CI]: - 134, - 100 mL; *P* < 0.001) and SGRQ score (+ 6.42 units; 95% CI: 5.40, 7.45; *P* < 0.001), and had higher risk of exacerbations (hazard ratio [HR]: 1.61 [95% CI: 1.50, 1.72]; *P* < 0.001) and all-cause mortality (HR: 1.41 [95% CI: 1.15, 1.72]; *P* < 0.001). Similar risks post-CID were observed in ECLIPSE.

**Conclusions:**

A CID within 6–12 months of follow-up was consistently associated with increased long-term risk of exacerbations and all-cause mortality, and predicted sustained meaningful loss in FEV_1_ and health status amongst survivors.

**Trial registration:**

NCT00268216; NCT00292552.

**Electronic supplementary material:**

The online version of this article (10.1186/s12931-018-0928-3) contains supplementary material, which is available to authorized users.

## Introduction

The heterogeneous and progressive nature of chronic obstructive pulmonary disease (COPD) has prompted interest in developing reliable measurements of its progression beyond mean decline rates in forced expiratory volume in 1 s (FEV_1_) [1–3]. Improvement in FEV_1_ is regularly assessed in clinical trials and has been shown to correlate with improvements in symptoms, health status [4, 5], and reductions in exacerbation rates [6, 7]. However, FEV_1_ alone is not sufficient to evaluate the longitudinal changes in the severity of COPD at an individual level [8], and other outcomes are required to monitor disease activity [9].

Recently, a composite measure of deterioration has been developed, comprising: lung function (≥100 mL decline in FEV_1_), health status (≥4-unit increase in St George’s Respiratory Questionnaire [SGRQ]), and the incidence of a moderate/severe exacerbation [10]. The thresholds for FEV_1_ and SGRQ were chosen as the accepted minimum clinically important differences (MCID) for these outcomes [11, 12]. The occurrence of one or more of these events was considered a clinically important deterioration (CID). Several short-term COPD studies have employed this endpoint as an a priori and post hoc measure of instability [10, 13–18]. In short-term trials, incremental gains in the prevention of short-term CID have been shown when comparing mono- and dual-bronchodilator therapy with placebo, between dual- and mono-bronchodilator therapy, and with dual-bronchodilator or triple therapy versus ICS/LABA therapy [10, 13–18]; however, the relationship between short-term CID and long-term outcome is unknown.

Management of COPD has moved towards a patient-centered approach, with regular monitoring of the adequacy of care, with the goal of matching appropriate therapy to patients who will benefit most effectively from them [3]. Understanding the association between short-term disease worsening and long-term outcomes would help physicians to better personalize pharmacological treatment at an early stage of a patient’s disease.

This post hoc analysis used data from a 3-year interventional study (TOwards a Revolution in COPD Health [TORCH]) [19] and a 3-year observational study (Evaluation of COPD Longitudinally to Identify Predictive Surrogate End-points [ECLIPSE]) [8, 20] to assess whether worsening of COPD, as measured by the presence of any component of the composite CID during the first 6 to 12 months, was a predictor of poor medium- to long-term outcome after a further 30 to 24 months of follow-up, respectively. Analysis of the TORCH data constituted the principal analysis as the presence of CID could be assessed earlier, at 6 months. In the ECLIPSE study CID could only be assessed at 12 months, and so was used as a confirmatory analysis to see if the prognostic findings of the TORCH study could be replicated.

## Methods

### Study design and treatments

The TORCH study (SCO30003, NCT00268216) was a double-blind, placebo-controlled, randomized study that assessed spirometric values, health status, frequency of exacerbations, and mortality in patients randomized to fluticasone propionate/salmeterol (FP/SAL) 500/50 μg, FP 500 μg, SAL 50 μg, or placebo [19]. The ECLIPSE study (SCO104960, NCT00292552) was a large, observational, longitudinal study of patients with moderate-to-very-severe COPD aimed at defining COPD phenotypes and identifying biomarkers to help predict disease progression [8, 20]. Detailed methodologies for both studies have been previously published [8, 19–21]. Both studies were conducted in accordance with the Declaration of Helsinki and good clinical practice guidelines, and approved by the relevant ethics review committees. All participants gave written informed consent.

### Patients

Patients with a diagnosis of COPD in the TORCH study were aged 40–80 years, current or former smokers with ≥10 smoking pack-years, had a pre-bronchodilator FEV_1_ < 60% of predicted value and a pre-bronchodilator FEV_1_/forced vital capacity (FVC) ratio ≤ 0.70. Exclusion criteria included a diagnosis of asthma or respiratory disorders other than COPD, lung-volume reduction or lung transplant surgery, and receiving long-term oral corticosteroid therapy.

In the ECLIPSE study patients were aged 40–75 years, with baseline post-bronchodilator FEV_1_ < 80% of predicted value, baseline post-bronchodilator FEV_1_/FVC ≤0.70, and ≥ 10 smoking pack-years. Exclusion criteria included known respiratory disorders other than COPD, history of other significant inflammatory disease, COPD exacerbation ≤4 weeks prior to enrollment, and prior lung surgery.

### Outcomes and assessments

Assessment of post-bronchodilator FEV_1_, and SGRQ score, was performed at 24, 48, 72, 96, 120, and 156 weeks post-treatment in the TORCH study [19]. In the ECLIPSE study, post-bronchodilator FEV_1_ was assessed at 3, 6, 12, 18, 24, 30, and 36 months after treatment, COPD-specific version of the SGRQ (SGRQ-C) was assessed annually, and exacerbations were assessed throughout the study. All-cause mortality and the frequency of moderate or severe exacerbations were assessed in both studies.

Composite CID was defined as any one of the following component events: (i) decrease of ≥100 mL from baseline in post-bronchodilator FEV_1_ [11]; (ii) increase of ≥4 units in SGRQ score from baseline [12]; (iii) incidence of a moderate/severe exacerbation (acute worsening of COPD requiring oral corticosteroids, antibiotics, emergency department treatment, or hospitalization).

Patients were categorized into two subgroups: (i) those who met ≥1 CID criteria within the first 6 months (TORCH) after enrollment (CID+), and (ii) those who did not (CID-). In the confirmatory analysis using ECLIPSE data, patient subgroups with (CID+) and without (CID-) a composite CID were defined within the first 12 months of enrollment. CID2+ and CID3+ patients were individuals with two or all three CID component deteriorations occurring at the assessment time, respectively.

Outcomes were assessed by CID status (CID+ or CID-) in a combined analysis, irrespective of which treatment patients were randomized to in the TORCH study.

### Statistical analysis

Since this was an analysis of the effect of changes in clinical outcomes (irrespective of treatment), analyses of TORCH study data were performed on the full intent-to-treat (ITT) population, with all treatment groups combined. Mean changes from baseline in post-bronchodilator FEV_1_ or SGRQ scores were compared (CID+ group vs CID- group) using a repeated measures model (least squares [LS] mean) including covariates of post-bronchodilator FEV_1_ deterioration status at Week 24, smoking status, geographical region, baseline FEV_1_ or SGRQ, time in weeks, week-by-baseline, and week-by-deterioration status at Week 24. Time to first moderate/severe or severe exacerbation and mortality in the 130 weeks after Week 24 were analyzed using Cox proportional hazards model with covariates of deterioration status at Week 24, smoking status at screening, and geographical region. Hazard ratios (HR) and confidence intervals (CI) were derived for the CID+ versus CID- group.

All comers were included in the ECLIPSE study data analysis, irrespective of physician choice of management. Statistical comparisons in LS mean change from baseline in post-bronchodilator FEV_1_ or SGRQ score for the CID+ versus CID- group were obtained from a repeated measures model including covariates of deterioration status at Month 12, smoking status at screening, geographical region, age, gender, baseline post-bronchodilator FEV_1_ or SGRQ, week, week-by-baseline, and week-by-deterioration status at Month 12. For the composite endpoint, FEV_1_ and SGRQ data were only used from common time points. Time to first moderate/severe or severe exacerbation and mortality in the 24 months after Month 12 were analyzed using Cox proportional hazards model with covariates of deterioration status at 12 months, smoking status at screening, geographical region, sex, and age. HRs and CIs were derived for CID+ versus CID- group.

In both studies, baseline and demographic data were generated using descriptive statistics, presented as mean (standard deviation [SD]) and n values (%).

## Results

### Patients

**Fig. 1:**
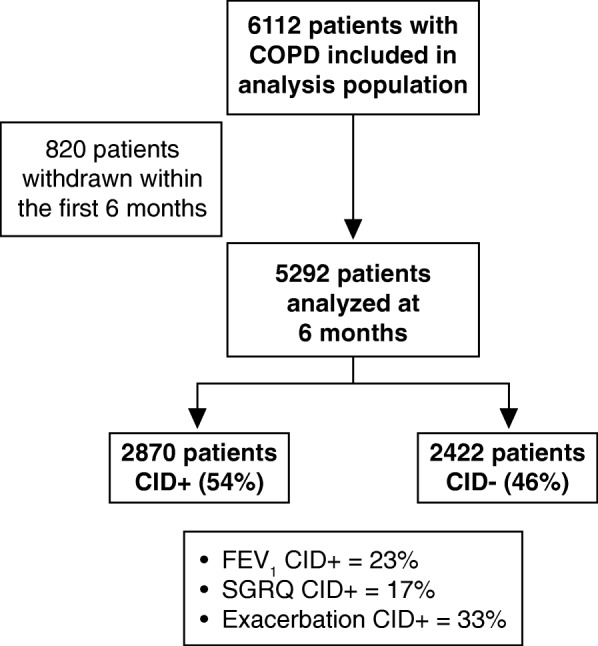
Proportions of patients experiencing CIDs in the TORCH study. CID, clinically important deterioration; CID+, presence of a CID within 6 months of enrollment into the study; CID-, absence of a CID within 6 months of enrollment into the study; COPD, chronic obstructive pulmonary disease; FEV_1_, forced expiratory volume in 1 s; SGRQ, St George’s Respiratory Questionnaire; TORCH, TOwards a Revolution in COPD Health

In the TORCH study, the ITT population included 2870 patients in the CID+ group and 2422 patients in the CID- group (Fig. 1). The most and least common cause of CID in the first 6 months was exacerbation (33%) and deterioration in SGRQ (17%), respectively (Fig. 1). Demographics and baseline characteristics were generally similar in the CID+ and CID- groups, although the proportion of female patients, patients with ≥2 previous exacerbations, and patients receiving inhaled corticosteroids (ICS) in the 12 months prior to the run-in period was greater in the CID+ compared with the CID- group (Table 1).

**Table 1:** Patient demographics and baseline characteristics (TORCH study; ITT population)

	CID+ population (*N* = 2870)	CID- population (*N* = 2422)	Total (*N* = 5292)
Age (years), mean (SD)	64.7 (8.2)	65.1 (8.4)	64.9 (8.3)
Male, *n* (%)	2151 (75)	1895 (78)	4046 (76)
BMI, kg/m^2^, mean (SD)	25.3 (5.3)	25.5 (5.2)	25.4 (5.2)
Current smoker at screening, *n* (%)	1277 (44)	1072 (44)	2349 (44)
Post-bronchodilator % predicted FEV_1_, mean (SD)	44.7 (13.8)	44.8 (12.8)	44.7 (13.4)
Patients receiving ICS in the 12 months prior to the run-in period, *n* (%)	1455 (51)	1037 (43)	2492 (47)
Previous exacerbations, *n* (%)			
0	1122 (39)	1164 (48)	2286 (43)
1	711 (25)	617 (25)	1328 (25)
≥ 2	1037 (36)	641 (26)	1678 (32)

*BMI* body mass index, *CID* clinically important deterioration, *CID+* presence of a CID within 6 months of enrollment into the study, *CID-* absence of a CID within 6 months of enrollment into the study, *ICS* inhaled corticosteroid, *ITT* intent-to-treat, *SD* standard deviation, *TORCH* TOwards a Revolution in COPD Health

The ECLIPSE study comprised 1442 patients in the CID+ group and 531 patients in the CID- group (Additional file 1: Figure S1; online data supplement). Demographics and baseline characteristics were generally similar in the CID+ and CID- groups, with the differences following the same pattern as for the TORCH study (Additional file 2: Table S1; online data supplement).

### Changes in lung function and health status over time based on CID status (TORCH study)

**Fig. 2:**
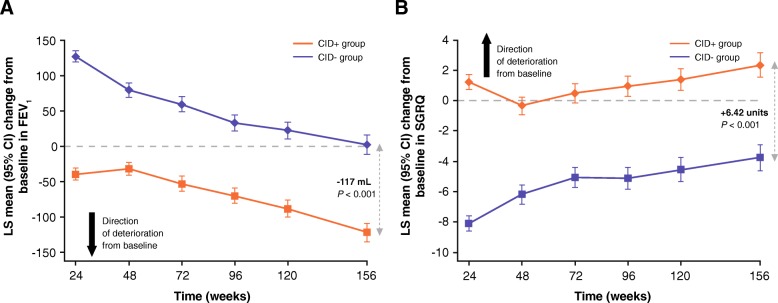
LS mean change from baseline in (**a**) FEV_1_ (mL) and (**b**) SGRQ score over time based on CID status (TORCH study ITT population)**,** CI, confidence interval; CID, clinically important deterioration; CID+, presence of a CID within 6 months of enrollment into the study; CID-, absence of a CID within 6 months of enrollment into the study; COPD, chronic obstructive pulmonary disease; FEV_1_, forced expiratory volume in 1 s; ITT, intent-to-treat; LS, least squares; SGRQ, St George’s Respiratory Questionnaire; TORCH, TOwards a Revolution in COPD Health

Post-bronchodilator FEV_1_ decreased in both CID groups at a similar rate from Week 24 to the end of follow up (Fig. 2a). Patients free of all CID events (CID- group) had an improvement in FEV_1_ at 24 weeks that returned to baseline at 156 weeks (Fig. 2a). By contrast, patients in the CID+ group showed a modest mean deterioration in FEV_1_ at Week 24, which was clinically important at 156 weeks (between-group difference: - 117 mL, 95% CI: -134, - 100; *P* < 0.001; Fig. 2a). At the 3-year time point, the odds of a deficit in lung function of ≥100 mL or ≥ 200 mL from baseline were approximately two-fold higher in the CID+ compared with the CID- group (Table 2).

**Table 2:** Odds/Risk of long-term adverse outcomes based on CIDs in the first 6 months of the TORCH study (ITT population)

Outcome	CID+ population (*N* = 2870)	CID- population (*N* = 2422)	CID+ vs CID-, OR (95% CI)
≥100 mL decrease in FEV_1_^a^, *n* (%)	2004 (70)	1296 (54)	2.00 (1.78, 2.24)*
≥200 mL decrease in FEV_1_^a^, *n* (%)	1696 (59)	1018 (42)	1.97 (1.77, 2.20)*
≥4-unit increase in SGRQ^a^, *n* (%)	1516 (67)	930 (53)	1.82 (1.60, 2.07)*
≥8-unit increase in SGRQ^a^, *n* (%)	1342 (59)	827 (47)	1.65 (1.45, 1.88)*
Moderate-to-severe COPD exacerbation^b^, *n* (%)	2082 (73)	1450 (60)	1.61^c^ (1.50, 1.72)*
Hospitalization events^b^, *n* (%)	797 (28)	491 (20)	1.55^c^ (1.38, 1.73)*
Risk (HR) of all-cause mortality^b^, *n* (%)	237 (8)	160 (7)	1.41^c^ (1.15, 1.72)*

*P < 0.001, ^a^risk of deterioration at 36-months assessed from the pre-randomization period; ^b^risk of a new exacerbation or all-cause death assessed from Week 24; ^c^data report HR (95% CI)

*CID* clinically important deterioration, *CID+* presence of a CID within 6 months of enrollment into the study, *CID-* absence of a CID within 6 months of enrollment into the study, *FEV*_*1*_ forced expiratory volume in 1 s, *HR* hazard ratio, *ITT* intent-to-treat, *OR* odds ratio, *SGRQ* St George’s Respiratory Questionnaire, *TORCH* TOwards a Revolution in COPD Health

Patients in the CID- group had clear improvement in health status at Week 24, with a decrease in SGRQ score from baseline of approximately 8 units; Fig. 2b. In the CID+ group, patients showed an increase from baseline in SGRQ score of 1.33 units (representing a worsening health status) at Week 24 (Fig. 2b). At study end, the difference in SGRQ score in the CID+ versus CID- group remained above the MCID (+ 6.42 units, 95% CI: 5.40, 7.45; *P* < 0.001; Fig. 2b). Patients in the CID+ group had greater odds of having a ≥ 4-unit and ≥ 8-unit worsening in SGRQ score from baseline at 36 months compared with those in the CID- group (Table 2).

### Future risk of exacerbations and all-cause mortality based on CID status (TORCH study)

**Fig. 3:**
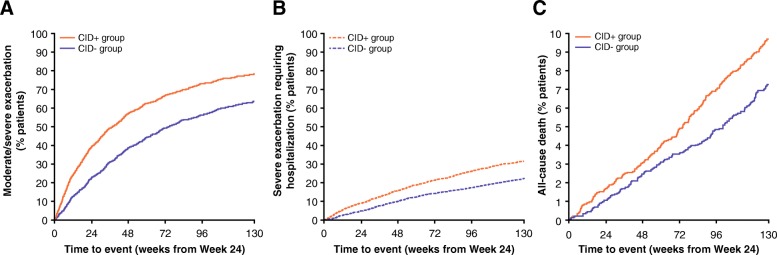
Time to first (**a**) moderate/severe exacerbation, (**b**) exacerbation requiring hospitalization, and (**c**) all-cause mortality, based on CID status (TORCH study; ITT population). CI, confidence interval; CID, clinically important deterioration; CID+, presence of a CID within 6 months of enrollment into the study; CID-, absence of a CID within 6 months of enrollment into the study; ITT, intent-to-treat; TORCH, TOwards a Revolution in COPD Health

Patients in the CID+ group had a significantly higher risk of exacerbations compared with patients in the CID- group. This pattern was observed for both moderate/severe exacerbations (HR [CID+ vs CID-]: 1.61; 95% CI: 1.50, 1.72; *P* < 0.001; Fig. 3a) and severe exacerbations requiring hospitalization (HR [CID+ vs CID-]: 1.55; 95% CI: 1.38, 1.73; *P* < 0.001; Fig. 3b). Median time to first moderate-to-severe exacerbation in the CID- group was 520 days compared with 265 days in the CID+ group. Patients in the CID+ group had a 41% increased risk of all-cause mortality compared with those in the CID- group (HR [CID+ vs CID-]: 1.41; 95% CI: 1.15, 1.72%; *P* < 0.001; Fig. 3c; Table 2).

### Comparison of CID type and frequency with all-cause mortality (TORCH study)

**Fig. 4:**
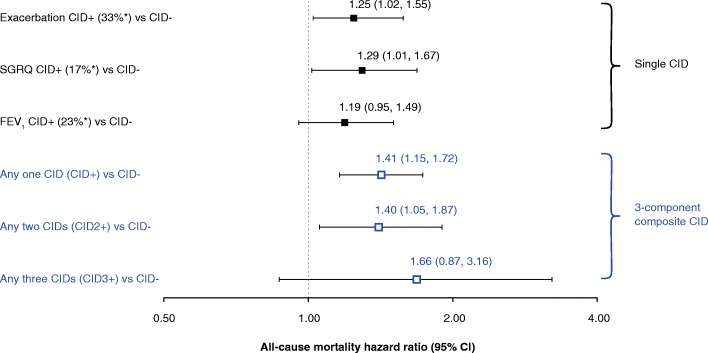
All-cause mortality hazard ratios of single CID events and composite CID events for CID+ patients versus CID- patients (TORCH study; ITT population)**.** *Proportion of patients with the specified CID in the first 6 months. CI, confidence interval; CID, clinically important deterioration; CID+, presence of a CID within 6 months of enrollment into the study; CID-, absence of a CID within 6 months of enrollment into the study; COPD, chronic obstructive pulmonary disease; FEV_1_, forced expiratory volume in 1 s; ITT, intent-to-treat; SGRQ, St George’s Respiratory Questionnaire; TORCH, TOwards a Revolution in COPD Health

Worsening of each component of the CID was associated with increased mortality risk by a broadly similar magnitude (19–29%) compared with absence of that CID event type; however, this was only statistically significant for the exacerbations and SGRQ components (Fig. 4). Freedom from all CIDs was associated with lower mortality risk than freedom from just one CID component alone (SGRQ or exacerbations or FEV_1_), indicating that each CID component contributed to the overall mortality risk. However, as so few patients had multiple types of CID events in the first 6-months, there remains some uncertainty in defining a CID-dose effect (Fig. 4).

### Predictions of long-term outcomes based on individual component deteriorations (TORCH study)

Individual CID event types within the first 6 months were associated with a greater risk of deterioration in that outcome at 3 years. For example, the change from baseline in FEV_1_ after 36 months was - 193 mL (95% CI: -213, - 172) for patients who had an FEV_1_ CID event in the first 6 months, compared with - 51 mL (95% CI: -74, - 27) and - 38 mL (95% CI: -57, - 20) for patients with a SGRQ and exacerbation CID event in the first 6 months, respectively (Additional file 2: Table S2; online data supplement). Similarly, SGRQ and exacerbation CID events led to the greatest risk of future deteriorations in health status and exacerbations, respectively (Additional file 2: Table S2; online data supplement).

FEV_1_ CID events or exacerbation CID events by 6 months had similar long-term impacts on health status at 36 months (+ 3.56 units [95% CI: 2.31, 4.80] and + 3.70 units [95% CI: 2.56, 4.83], respectively; Additional file 2: Table S2; online data supplement).

### Analysis of the ECLIPSE study data

**Fig. 5:**
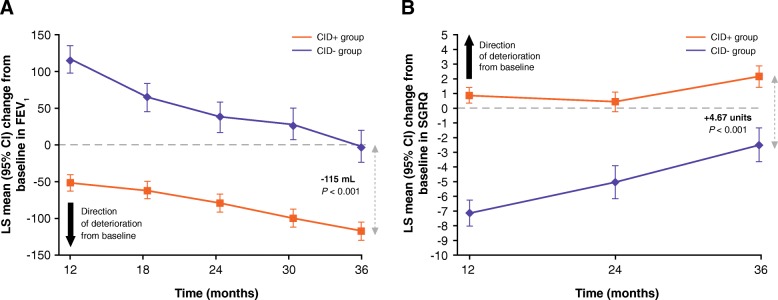
LS mean change from baseline in (**a**) FEV_1_ (mL) and (**b**) SGRQ score over time based on CID status (ECLIPSE study). CI, confidence interval; CID, clinically important deterioration; CID+, presence of a CID within 12 months of enrollment into the study; CID-, absence of a CID within 12 months of enrollment into the study; COPD, chronic obstructive pulmonary disease; ECLIPSE, Evaluation of COPD Longitudinally to Identify Predictive Surrogate End-points; FEV_1_, forced expiratory volume in 1 s; LS, least squares; SGRQ, St George’s Respiratory Questionnaire

In the ECLIPSE study, changes in FEV_1_ and SGRQ between the CID+ and CID- groups were similar to those observed in the TORCH study (Fig. 5a and Fig. 5b, respectively). At study end, the difference in mean change from baseline in FEV_1_ was - 115 mL (95% CI: -141, - 89; *P* < 0.001) and the difference in mean change from baseline SGRQ was + 4.67 units (95% CI: 3.32, 6.02; *P* < 0.001).

**Fig. 6:**
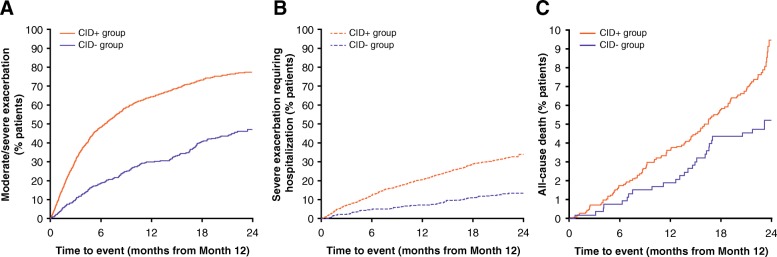
Time to first (**a**) moderate/severe exacerbation, **b** exacerbation requiring hospitalization, and (**c**) all-cause mortality, based on CID status (ECLIPSE study)**.** CID, clinically important deterioration; CID+, presence of a CID within 12 months of enrollment into the study; CID-, absence of a CID within 12 months of enrollment into the study; ECLIPSE, Evaluation of COPD Longitudinally to Identify Predictive Surrogate End-points

Compared with patients in the CID- group, those in the CID+ group had a significantly higher risk of moderate/severe exacerbations (HR: 2.54; 95% CI: 2.20, 2.93; *P* < 0.001; Table 3; Fig. 6a), exacerbations requiring hospitalization (HR: 2.81; 95% CI: 2.17, 3.63; *P* < 0.001; Table 3; Fig. 6b), and all-cause mortality (HR: 1.59; 95% CI: 1.04, 2.41; *P* = 0.031; Table 3; Fig. 6c).

**Table 3:** Risk of long-term adverse outcomes based on CIDs in the first 12 months of the ECLIPSE study

Outcome within 130 weeks of follow-up	CID+ population (*N* = 1442)	CID- population (*N* = 531)	CID+ vs CID-, HR (95% CI)
Moderate/severe COPD exacerbation, *n* (%)	1082 (75)	232 (44)	2.54 (2.20, 2.93)*
Hospitalization events, *n* (%)	454 (31)	66 (12)	2.81 (2.17, 3.63)*
Risk (HR) of all-cause mortality, *n* (%)	121 (8)	27 (5)	1.59 (1.04, 2.41)†

*P < 0.001; ^†^*P* = 0.031

*CID* clinically important deterioration, *CID+* presence of a CID within 6 months of enrollment into the study, *CID-* absence of a CID within 6 months of enrollment into the study, *ECLIPSE* Evaluation of COPD Longitudinally to Identify Predictive Surrogate End-points, *FEV*_*1*_ forced expiratory volume in 1 s, *HR* hazard ratio, *ITT* intent-to-treat, *SGRQ* St George’s Respiratory Questionnaire

## Discussion

This exploratory analysis evaluated whether CID status (a composite measure of early deterioration in COPD), assessed after the first 6 months of observation, could be used to predict a range of medium- to long-term patient outcomes after 3 years of follow-up. Patients who experienced a CID event in the first 6–12 months were found, in two separate 3-year studies (TORCH and ECLIPSE), to have consistently worse long-term outcomes, including sustained and important loss of FEV_1_ and health status, and a greater risk of exacerbation and mortality, compared with more stable patients free of short-term CID events [11, 12].

Disease progression in COPD is not confined to decline in FEV_1_. Medium-term deterioration in patients with COPD has been reported in terms of SGRQ score [22], six-minute walking distance [23] and daily physical activity [24]. The correlation between changes in these different measures of severity is not strong, as reported, for example, between FEV_1_ and SGRQ [4, 25], exacerbations and FEV_1_ [26] and physical activity, FEV_1_ and SGRQ [24]. As no single measure appears to capture all aspects of worsening COPD reliably in all patients, there is a strong argument for a new composite measure.

A composite CID assessment of multifactorial deterioration within 6 months of randomization in the TORCH study was shown to predict an increase in the risk of mortality to a greater extent than any single component of deterioration (eg, exacerbations). Patients free of any CID component within the first 6 months of the TORCH study had the lowest mortality risk, even compared with those experiencing only one CID component. Therefore, it would appear short-term deterioration assessed by a composite CID may predict all-cause mortality, long term. This has also been shown in a post hoc analysis of the Understanding Potential Long-Term Impacts on Function with Tiotropium (UPLIFT; NCT00144339) study, which used the same definition of CID as that presented here [27]. Furthermore, a recent analysis focused on the subgroup of patients who received the most intensified treatment in the TORCH study (FP/SAL 500/50 μg) showed similar levels of sustained loss in lung function and health status, and similarly increased risk of exacerbation and all-cause mortality in CID+ versus CID- patients compared with those presented here [28], indicating that the predictive power of on-treatment CID is not influenced by that same treatment after the CID event. Additionally, the presence of a composite CID was able to predict an increased risk of deterioration in all of the three CID domains at 3 years, supporting our rationale for using a combined measure of short-term deterioration, rather than a single measure. The predictive power of CID status in each study was evident (at 6 months in TORCH and 12 months in the ECLIPSE study). In both studies, after 36 months, the difference in change from baseline between the CID+ and CID- groups for both FEV_1_ and SGRQ score was statistically significant and exceeded the MCID for those outcome measures [11, 12]. Importantly, CID status also predicted all-cause mortality in both studies; an outcome that did not form part of the composite measure.

Although the cumulative 3-year outcomes were worse for the CID+ than the CID- group, the subsequent rate of further decline in the CID measures, beyond the assessment period (6 months for TORCH and 12 months for ECLIPSE), was similar for the two groups. Minimal recovery was observed following an initial CID, indicating that these events could signify permanent loss in health status and lung function.

Guidelines advocate regular monitoring of the adequacy of COPD care in individual patients looking at exacerbations, symptom and lung function to prevent disease progression [3]. However, no formal framework for such monitoring has been advocated. We believe indicators of increased disease activity based on a lack of favorable treatment response across the CID event types may have utility and prognostic value. The composite CID presented in this exploratory analysis appears to be a potential tool to identify increased disease activity and predict poor long-term outcome in patients with COPD based on short-term deterioration on treatment [10, 16]. While other researchers have suggested that a lack of stability in lung function or health status could predict increased future risk in COPD [6, 29], to our knowledge, the use of a prognostic composite endpoint of treatment response containing lung function, health status, and exacerbation frequency, has not previously been reported. This composite CID captures the heterogeneous nature of COPD by incorporating relatively short-term clinical measures of these three different COPD outcomes. Using a three-component composite measure of deterioration in COPD ensures that variability in a patient’s response to therapy and potential for deterioration is fully captured. In concordance with this view, a recent composite CID analysis, highlighted little or no statistical concordance between the different component measures of deterioration, utilized in a 1-year exacerbation study [13].

Limitations of this analysis included its post hoc nature and the greater number of patients in the CID+ group versus the CID- group with a baseline history of exacerbations and who received ICS prior to the study in both trials, which could influence their likelihood of having an exacerbation CID. This difference did not impact long-term outcomes post-CID in this study, with prognostic findings in previous analyses of TORCH also demonstrating similar results across all treatment arms [28, 30, 31]. The CID concept developed here is in some respects opportunistic as it utilizes outcome measures available in existing clinical trial data sets. However, it includes three domains of COPD progression that can be measured relatively easily in large numbers of patients, unlike exercise capacity and daily physical activity which are more difficult to measure. Another potential limitation concerns two of the measures that form the composite CID endpoint. The complexity of post-bronchodilator rather than pre-bronchodilator FEV_1_ measurement, and use of the long and complicated SGRQ rather than the simpler COPD Assessment Test (CAT), which also has an MCID [32, 33], will restrict their use to clinical trials. However, whilst the TORCH study focused on post-bronchodilator FEV_1_, our analysis of the ECLIPSE study data used both post- and pre-bronchodilator FEV_1_ values in the CID assessment, providing concordant findings (data not shown). Furthermore, this analysis was a test of principle and prospective studies will confirm the use of these measurements and test whether use of other outcomes, like CAT, might be more applicable to routine practice, or may replace SGRQ in assessing deterioration in health status [17, 34, 35].

Another aspect of this analysis determined by the primary trial datasets was the time and frequency at which data for all three components was available. The earliest assessment of a CID was dictated by the availability of data at 6 and 12 months in the TORCH and ECLIPSE studies, respectively. It remains unclear whether short-term assessment of a CID could be made earlier than assessed in either of these studies. It is possible that using more frequent CID assessments of FEV_1_ and health status, rather than a single time point assessment as employed here, can provide more accurate prognostic assessments of mortality risk for each of these individual component event types, as suggested in a recent post hoc analysis of the UPLIFT trial [36]. As prognostic assessments in several landmark studies have identified concordant increased risk of exacerbations requiring hospitalization and mortality post CID in populations not enriched for exacerbation risk [27, 36, 37], we propose future prospective studies designed to monitor CID events in a broader symptomatic patient cohort with regular 3 monthly visits could have similar or greater predictive ability. This will be of importance to test whether less severely affected patients who are at high risk of poor medium-term outcomes can be identified and more optimally treated. These questions are being investigated prospectively in ongoing studies.

This analysis tested the predictive validity of the CID, an event-based, categorical measure of a patient’s state. Previous analyses have tested the utility of CID as a measure of treatment efficacy and the benefit of escalation in therapy from mono- to dual-bronchodilation, dual-bronchodilation compared with ICS/LABA and triple therapy versus ICS/LABA therapy [10, 13–18]. All three components of the CID may find application in clinical trials, however perhaps its greatest potential may lie in predicting long-term response to treatment. Currently this requires the use of trials that are typically of 3 years’ duration that may introduce bias due to differential dropout of patients with more severe disease [38]. A validated surrogate marker of medium-term outcome, such as CID status, may allow shorter term trials to test disease modification or more reliably identify candidate treatments for long-term trials.

## Conclusions

The results of this retrospective analysis of two 3-year COPD studies demonstrate that short-term deterioration assessed using a composite CID endpoint had prognostic value in identifying patients with increased future risk of a sustained loss of lung function and health status, and importantly increased incidence of exacerbations requiring hospitalization and all-cause mortality. This concept of evaluating short-term changes across multiple endpoints may be a useful tool for physicians in identifying patients at greater risk of worsening of COPD and in designing trials to test treatments that may mitigate that risk.

## Additional files

Additional file 1: **Figure S1.** Proportions of patients experiencing CIDs in the ECLIPSE study. (EPS 1962 kb)

Additional file 2: **Table S1.** Patient demographics and baseline characteristics (ECLIPSE study). **Table S2.** Long-term outcomes based on single-component short-term CIDs (TORCH study; ITT population). (DOCX 110 kb)

## Abbreviations

BMI: Body mass index
CAT: COPD Assessment Test
CI: Confidence intervals
CID: Clinically important deterioration
COPD: Chronic obstructive pulmonary disease
ECLIPSE: Evaluation of COPD Longitudinally to Identify Predictive Surrogate End-points
FEV_1_: Forced expiratory volume in 1 s
FP: Fluticasone propionate
FVC: Forced vital capacity
HR: Hazard ratio
ICS: Inhaled corticosteroids
ITT: Intent-to-treat
LABA: Long-acting β adrenoceptor agonists
LS: Least squares
MCID: Minimum clinically important difference
OR: Odds ratio
SAL: Salmeterol
SD: Standard deviation
SGRQ: St George’s respiratory questionnaire
TORCH: Towards a Revolution in COPD Health
UPLIFT: Understanding Potential Long-Term Impacts on Function with Tiotropium
