# Supplementary material for: Long-term outcomes following first short-term clinically important deterioration in COPD
Source: Respir Res. 2018 Nov 20;19:222. doi: 10.1186/s12931-018-0928-3 (PMC6245880; doi:10.1186/s12931-018-0928-3)
Supplement: Supplementary file 2 — Table S1. Patient demographics and baseline characteristics (ECLIPSE study). Table S2. Long-term outcomes based on single-component short-term CIDs (TORCH study; ITT population). (DOCX 110 kb) [file 12931_2018_928_MOESM2_ESM.docx]

# Additional file 2

**Table S1** Patient demographics and baseline characteristics (ECLIPSE study)

|  | **CID+ population**  **(N=1442)** | **CID- population**  **(N=531)** | **Total**  **(N=1973)** |
| --- | --- | --- | --- |
| **Age (years) mean (SD)** | 63.5 (6.99) | 63.0 (7.18) | 63.4 (7.04) |
| **Male, n (%)** | 923 (64) | 361 (68) | 1284 (65) |
| **BMI, kg/m^2^, mean (SD)** | 26.4 (5.5) | 27.1 (6.1) | 26.6 (5.7) |
| **Current smoker at screening, n (%)** | 511 (35) | 194 (37) | 705 (36) |
| **Post-bronchodilator % predicted FEV_1_, mean (SD)** | 47.7 (15.7) | 51.2 (15.4) | 48.7 (15.7) |
| **ICS users at screening, n (%)** | 1122 (78) | 324 (61) | 1446 (73) |
| **Previous exacerbations (%)** |  |  |  |
| **0** | 657 (46) | 383 (72) | 1040 (53) |
| **1** | 404 (28) | 99 (19) | 503 (25) |
| **2** | 215 (15) | 29 (5) | 244 (12) |
| **≥3** | 166 (12) | 20 (4) | 186 (9) |

BMI, body mass index; CID, clinically important deterioration; CID+, presence of a CID within 6 months of enrollment into the study; CID-, absence of a CID within 6 months of enrollment into the study; ECLIPSE, Evaluation of COPD Longitudinally to Identify Predictive Surrogate End-points; FEV_1_, forced expiratory volume in 1 second; ICS, inhaled corticosteroid; SD, standard deviation

**Table S2** Long-term outcomes based on single-component short-term CIDs (TORCH study; ITT population)

|  | **Short-term CID type (in first 6 months) CID+ vs CID- status** | | | |
| --- | --- | --- | --- | --- |
| **Outcome after 36 months** | **Composite CID** | **FEV_1_ CID only** | **SGRQ CID only** | **Exacerbation CID only** |
| **Change in FEV_1_/mL^a^** | -117 (-134, -100)* | -193 (-213, -172)* | -51 (-74, -27)* | -38 (-57, -20)* |
| **Change in SGRQ score/SGRQ units^a^** | +6.42 (5.40, 7.45)* | +3.56 (2.31, 4.80)* | +10.14 (8.91, 11.38)* | +3.70 (2.56, 4.83)* |
| **Change in exacerbation rate^b^** | 1.61 (1.50, 1.72)* | 1.02 (0.95, 1.11) | 1.12 (1.02, 1.22)^†^ | 2.21 (2.06, 2.36)* |

**P* < 0.001; ^†^*P* < 0.05

^a^LS mean (95% CI) change from baseline at 36 months; ^b^HR (95% CI) for exacerbations 6–36 months from randomization.

CI, confidence interval; CID, clinically important deterioration; CID+, presence of a CID within 6 months of enrollment into the study; CID-, absence of a CID within 6 months of enrollment into the study; COPD, chronic obstructive pulmonary disease; FEV_1_, forced expiratory volume in 1 second; ITT, intent-to-treat; SGRQ, St George’s Respiratory Questionnaire; TORCH, Towards a Revolution in COPD Health

**Figure S1** Proportions of patients experiencing CIDs in the ECLIPSE study


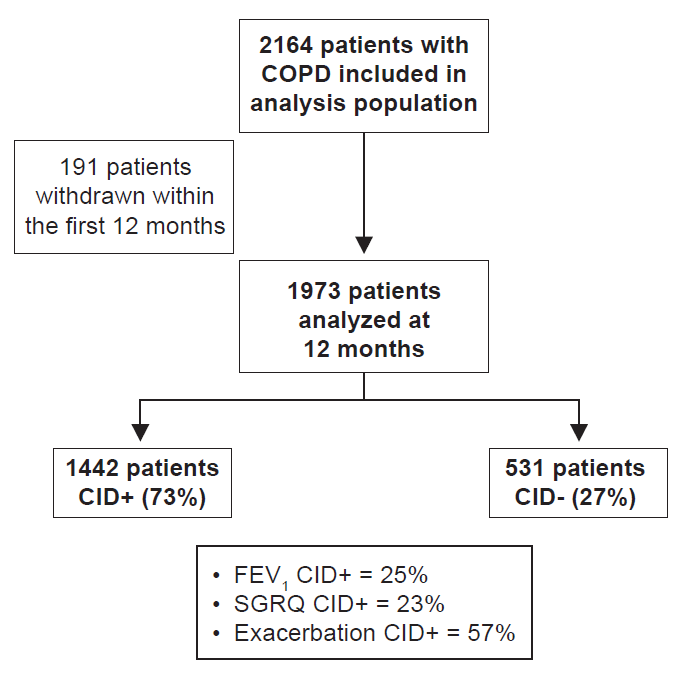
 CID, clinically important deterioration; CID+, presence of a CID within 12 months of enrollment into the study; CID-, absence of a CID within 12 months of enrollment into the study; COPD, chronic obstructive pulmonary disease; ECLIPSE, Evaluation of COPD Longitudinally to Identify Predictive Surrogate End-points; FEV_1_, forced expiratory volume in 1 second; SGRQ, St George’s Respiratory Questionnaire
